# Comment on ‘Cachexia in Preclinical Rheumatoid Arthritis: Longitudinal Observational Study of Thigh Magnetic Resonance Imaging From Osteoarthritis Initiative Cohort’ by Moradi Et Al.

**DOI:** 10.1002/jcsm.13590

**Published:** 2024-09-28

**Authors:** Liangping Zhang, Xizhuo Zhou, Gaoyong Jia

**Affiliations:** ^1^ Department of Orthopedics Hangzhou Linping District Hospital of Integrated Traditional Chinese and Western Medicine Hangzhou China; ^2^ Department of Orthopedics Zhejiang Chinese Medical University Hangzhou China; ^3^ Department of Orthopedics Hangzhou TCM Hospital Affiliated to Zhejiang Chinese Medical University (Hangzhou Hospital of Traditional Chinese Medicine) Hangzhou China

To the Editor:

We read with interest the recent publication in the *Journal of Cachexia, Sarcopenia and Muscle* by Moradi et al. ‘Cachexia in preclinical rheumatoid arthritis: Longitudinal observational study of thigh magnetic resonance imaging from osteoarthritis initiative cohort’ [1], which sheds light on the emerging evidence of cachexia in early rheumatoid arthritis stages. As healthcare professionals, we are heartened by the authors' focus on this significant issue, which brings attention to critical concerns in autoimmune disease and musculoskeletal health. This paper aims to commend the study's contributions while discussing areas necessitating cautious interpretation and further exploration.

Firstly, the study uses propensity score matching (PS matching) for data analysis. In comparison, the generalized estimating equation (GEE) model might be more suitable for this study. GEE can capture and analyse time‐varying effects and has advantages in analysing data on muscle composition and fat changes measured repeatedly at multiple time points in the same rheumatoid arthritis (RA) patients. It is suitable for studying trends over different time points and can provide robust parameter estimates.

Secondly, potential confounding variables. Although the article adjusts for several confounding factors (e.g., age, gender, body mass index and knee osteoarthritis status), there are still some potential confounding variables that may not have been fully considered. For example, participants' socioeconomic factors, physical activity levels, dietary habits, lifestyle factors (such as smoking, alcohol consumption and sleep quality) and other health conditions may significantly influence the progression and outcomes of RA [2]. Considering these factors can further enhance the robustness of the study results.

Thirdly, the impact of whole‐body and thigh muscle and fat distribution on RA development can be further explored through detailed subgroup analysis. Such analysis can help understand the specific effects of changes in whole‐body and regional muscle and fat distribution on overall metabolic health, providing a basis for personalized health management. Studies have shown that individuals with significant thigh muscle atrophy are more likely to experience increased disability and metabolic disorders [3]. While changes in muscle and fat distribution in other parts of the body also affect health, the specific impact of thigh muscle and fat distribution on overall health outcomes may differ from other regions, highlighting the importance of a comprehensive whole‐body approach [4].

Despite the authors' recommendations, some reflection may be necessary for us as healthcare professionals. The study emphasizes the importance of early detection and intervention in the preclinical rheumatoid arthritis (Pre‐RA) stage. The article highlights the importance of providing comprehensive medical support to these patients, addressing the multifaceted challenges posed by the disease through a range of support services. For Pre‐RA patients, healthcare professionals can guide them in utilizing medical and social resources, including comprehensive health assessments, personalized diet and exercise plans, and regular body composition analysis and inflammation biomarker testing. Additionally, healthcare professionals can provide education and counselling services to help patients understand Pre‐RA risk factors and prevention strategies, thereby improving their quality of life and long‐term health outcomes.

Finally, we appreciate the authors' innovative and insightful work in assessing muscle and fat changes in rheumatoid arthritis patients, and we hope our comments will inspire further discussion and more comprehensive evaluations in rheumatoid arthritis research.

## Conflicts of Interest

The authors declare no conflicts of interest.
